# Examining Predictors of Longevity Among Iowa Centenarians: A Latent Profile Analysis

**DOI:** 10.1093/geroni/igaf122.067

**Published:** 2025-12-31

**Authors:** Elnaz Abaei

**Affiliations:** Iowa State University, Ames, Iowa, United States

## Abstract

Longevity is influenced by a complex interplay of personal and social factors, yet few studies have examined the interrelationship of these factors. Using data from the Iowa Centenarian Study, we computed latent profile analysis (LPA), including 138 centenarians to identify distinct profiles based on four domains: 1. relationship variables: marital status, marriage length, marriage influence, and relationships with parents; 2. educational experiences: years of education and learning a foreign language; 3. social support: guidance, reliable alliance, reassurance of worth, social integration, attachment, and the opportunity to provide nurturance, and 4. NEO personality traits: neuroticism, extraversion, openness to experience, agreeableness, and conscientiousness. Analyses were conducted in two steps: first, within-domain solutions were identified, followed by a between-domain solution. Preliminary analysis identified marriage length, education, neuroticism, and attachment as key distinguishing factors. These variables were then used in a between-domain LPA, resulting in a two-class solution. Class 1 (Low Education, 81.2%) is characterized by moderate marriage length, lower education, higher neuroticism, and insecure attachment. Class 2 (Long-Term Bonds, 18.8%) is characterized by long-term marriages, higher education, lower neuroticism, and secure attachment. The mean group differences revealed significant group differences for marriage length, F(1,93)=7.234, p=.008, education, F(1,130)=39.507, p<.001, and attachment, F(1,110)=106.239, p<.001, while no significant effect was found for neuroticism, F(1,105)=2.162, p=.144. Applying LPA in this study offers a novel approach to identifying heterogeneous centenarians and insights into the diverse pathways to longevity. Practical implications include targeted interventions focused on relationship stability, education, and emotional well-being to promote longevity.

